# The incidence and risk factors of acute kidney injury after hepatobiliary surgery: a prospective observational study

**DOI:** 10.1186/1471-2369-15-169

**Published:** 2014-10-23

**Authors:** Eunjung Cho, Sun-Chul Kim, Myung-Gyu Kim, Sang-Kyung Jo, Won-Yong Cho, Hyoung-Kyu Kim

**Affiliations:** Division of Nephrology, Department of Internal Medicine, Korea University Anam Hospital, 5Ka, Anam-Dong, Sungbuk-Gu, Seoul, 136-705 Korea

**Keywords:** Acute kidney injury, Postoperative complications, Neutrophil gelatinase-associated lipocalin

## Abstract

**Background:**

Although intraperitoneal surgery is a major operation associated with postoperative acute kidney injury (AKI), the incidence, risk factors, and long-term renal outcome are not well known. We aimed to determine the risk factors and 6 months renal outcome in patients with clinical or subclinical AKI after hepatobiliary surgery. We also assessed the validity of urine neutrophil gelatinase-associated lipocalin (NGAL) in the early detection of AKI or prediction of renal outcome.

**Methods:**

This prospective observational study enrolled patients with normal renal function who underwent hepatobiliary surgeries. Urine and serum samples were collected for NGAL measurement.

**Results:**

Among 131 patients, 10 (7.6%) developed postoperative AKI. Urine NGAL at 12 h postsurgery was the most predictive parameter for the diagnosis of AKI (cutoff, 92.85 ng/mL). With the cutoff value, subclinical AKI was diagnosed in 42 (32.1%) patients. Patients with clinical AKI and those with subclinical AKI were assigned to the AKI group. The AKI group had significantly higher model for end-stage liver disease and sodium (MELD-Na) score, lower albumin level, and longer hospital stay after surgery than the non-AKI group. Older age and higher MELD-Na score were independent risk factors for the development of postoperative AKI. At 6 months postsurgery, the estimated glomerular filtration rate (eGFR) in the AKI group was significantly lower than that in the non-AKI group, although the baseline eGFR was not different. In multiple linear regression analysis, the maximum urine NGAL level during 24 h postsurgery, intraoperative fluid balance, and having liver transplantation were significantly associated with a poor 6 months renal outcome.

**Conclusion:**

Urine NGAL was useful in the early diagnosis of postoperative AKI as well as in predicting the 6 months renal outcome after hepatobiliary surgery. A considerable proportion of patients developed subclinical AKI, and these patients showed worse renal outcome compared with the non-AKI group.

**Electronic supplementary material:**

The online version of this article (doi:10.1186/1471-2369-15-169) contains supplementary material, which is available to authorized users.

## Background

Intraperitoneal surgery has been known as a common risk factor for postoperative acute kidney injury (AKI) along with cardiac surgery with cardiopulmonary bypass or vascular procedures with aortic cross-clamping. However, the incidence and outcome of AKI after an intraperitoneal procedure is largely unknown because most studies on postoperative AKI have been performed in patients undergoing cardiac surgeries or transplantation. In addition, the validity of new biomarkers in the early detection of AKI or in predicting outcome after intraperitoneal surgery is not known due to the paucity of data.

Since newly emerging biomarkers such as neutrophil gelatinase-associated lipocalin (NGAL) and interleukin-18 have been extensively validated as more sensitive early diagnostic markers for AKI than serum creatinine, the concept of subclinical AKI has recently emerged. Subclinical AKI can be defined as the presence of increased levels of renal tubular injury biomarkers without fulfilling the consensus criteria for AKI based on serum creatinine level or urine output [1]. Although a few studies demonstrated the poor outcome of patients who were classified as having subclinical AKI, the clinical significance of these patients further needs to be determined.

This study first assessed the incidence of postoperative clinical and subclinical AKI in patients with normal baseline renal function who underwent elective hepatobiliary surgeries, one of the most common intraperitoneal surgeries. Then, we compared the characteristics and outcomes between the AKI group (including patients with subclinical AKI) and the non-AKI group, and also determined the diagnostic and prognostic values of urine NGAL in this population.

## Methods

### Ethics statement

The study protocol was approved by the Institutional Review Board of Korea University Anam Hospital (IRB No. ED11094). Written informed consents were obtained from all patients.

### Patients and study design

Patients who planned to undergo elective hepatobiliary surgery in Korea University Anam Hospital were sequentially enrolled from July 2011 to January 2013. Patients <18 years of age, with baseline estimated glomerular filtration rate (eGFR) of <60 ml/min/1.73 m^2^, on maintenance renal replacement therapy (RRT), or who developed AKI preoperatively were excluded. Blood and urine samples were obtained before surgery (serum NGAL-Pre and urine NGAL-Pre). Urine samples were also obtained postoperatively at 6, 12, and 24 h (urine NGAL-6 h, NGAL-12 h, NGAL-24 h), and serum samples at 24 h (serum NGAL-24 h). Patients were followed up at the outpatient clinic after discharge, and serum creatinine levels were measured at 1, 3, and 6 months after the surgery.

Postoperative clinical AKI was diagnosed according to the Acute Kidney Injury Network (AKIN) criteria (i.e., ≥0.3 mg/dL or a ≥50% increase in the serum creatinine level from the baseline value within 48 h, or a urine output <0.5 mL/kg/hr for ≥6 h). Subclinical AKI was defined as an increase in the serum or urine biomarker (NGAL in this study) level above the determined cutoff value, without meeting the AKIN criteria [2–4]. This definition was also used in a multicenter pooled analysis by Hasse et al. and patients with subclinical AKI showed poorer outcome compared to non-AKI group who had no increase of both NGAL and serum creatinine [2]. The eGFR was estimated using the Modification of Diet in Renal Disease equation.

We first assessed the diagnostic ability of NGAL for the AKIN criteria-based clinical AKI. Then, we divided the patients into two groups: those with AKI (clinical or subclinical) and those without AKI. Baseline characteristics, surgical characteristics, and outcome variables, including death, length of hospital stay, and decrease of eGFR during the follow-up period, were compared between the two groups.

The primary outcome was the development of postoperative AKI, and the secondary outcomes were the length of hospitalization, postoperative death and the decrease in eGFR at 6 months after the surgery.

### Laboratory measurements

Collected blood and urine samples were centrifuged, and the supernatants were stored immediately at -80°C until the biomarker assay. Serum and urine NGAL were measured using NGAL ELISA kit (BioPorto, Gentofte, Denmark) according to the manufacturer’s instructions.

### Statistical analysis

SPSS software, version 19.0, was used for statistical analyses. Comparisons between the two groups were performed by the Student’s t-test or the Mann–Whitney U test for numerical data, and χ2 test or Fisher’s exact test for categorical data. To examine the diagnostic performance of urine NGAL for the development of clinical AKI, receiver operating characteristic (ROC) analysis was performed. We conducted multivariate logistic regression analysis to identify the risk factors for the development of clinical or subclinical AKI, and multiple linear regression analysis to identify the predictors for the extent of decrease in eGFR at 6 months postsurgery.

## Results

### Study population

During the study period, 135 patients, with informed consents, underwent elective hepatobiliary surgeries. Two patients who developed clinical AKI preoperatively and another two patients with a baseline eGFR of <60 ml/min/1.73 m^2^ were excluded. We enrolled 131 patients (84 male, 63.2%; mean age, 57.0 ± 12.4 years). The patients had the following underlying diseases: hepatocellular carcinoma (41.2%), liver metastasis of other primary cancers (20.6%), cholangiocarcinoma (14.5%), advanced liver cirrhosis for liver transplantation (6.9%), and others including intrahepatic duct stone and biliary cystadenoma. Fifty-five (42.0%) patients underwent hepatectomy alone; 56 (42.7%) underwent hepatectomy along with the resection of another organ, including gall bladder, diaphragm, inferior vena cava, or portal vein; and 20 (15.3%) underwent liver transplantation.

### Role of urine NGAL in the early detection of AKI and the incidence of subclinical AKI

**Figure 1:**
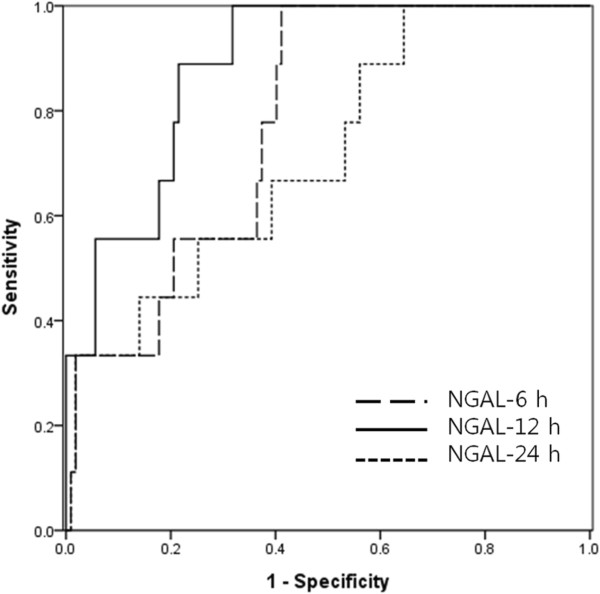
**Receiver operating characteristic (ROC) curves of urine NGAL for the diagnosis of clinical AKI.** The areas under the ROC curves are as follows: urine NGAL-6 h, 0.780 (95% CI 0.659-0.901, p = 0.005); urine NGAL-12 h, 0.886 (95% CI 0.803-0.969, p <0.001); urine NGAL-24 h, 0.714 (95% CI 0.549-0.880, p = 0.033).

Postoperative clinical AKI developed in 10 (7.6%) patients: five in AKIN stage 1, two in stage 2, and three in stage 3. And most (8 of 10) clinical AKI occurred in patients who had liver transplantation. None of the patients with clinical AKI had oliguria or required RRT. Table 1 presents the urine NGAL levels of patients with or without clinical AKI, and shows that the level increased significantly at 12 h postsurgery in patients with AKI compared with that in patients without AKI.The area under the ROC curve of urine NGAL-12 h for predicting clinical AKI was 0.886 (95% CI 0.803-0.969, p <0.001; cutoff 92.85 ng/mL, sensitivity 77.8%, and specificity 80.4%; Figure 1). With the use of this cutoff value and the maximum value of serial urine NGAL levels during 24 h postsurgery, we identified additional 42 (32.1%) patients who developed subclinical AKI and reclassified these patients into the AKI group together with patients who developed clinical AKI. Patients with subclinical AKI accounted for 80.8% (42 of 52) of the AKI group and 80.9% (34 of 42) of patients with subclinical AKI had non-transplant surgery.

**Table 1:** **Serum and urine NGAL levels in patients with or without clinical AKI**

NGAL (ng/mL)		Clinical AKI (n =10)	No AKI (n =121)	P
Serum	Pre	161.55 ± 150.69	167.05 ± 130.83	0.900
	24 h	491.78 ± 517.91	451.70 ± 385.93	0.771
Urine	Pre	39.67 ± 32.60	58.90 ± 156.90	0.700
	6 h	283.05 ± 350.69	83.51 ± 86.12	0.128
	12 h	374.20 ± 366.72	72.46 ± 134.34	0.039
	24 h	257.72 ± 273.39	98.74 ± 145.14	0.121

### Comparison of baseline and operation-related characteristics in patients with or without AKI

As shown in Table 2, there were no differences in age, sex, and the prevalence of hypertension, diabetes, or heart disease between the AKI and non-AKI groups. Baseline eGFR was >90 ml/min/1.73 m^2^ and similar in both groups. However, serum sodium and albumin levels were significantly lower, and total bilirubin level and prothrombin time (international normalized ratio [INR]) were significantly higher in the AKI group than in the non-AKI group. However, the levels of aspartate aminotransferase (AST) and alanine aminotransferase (ALT) were not different between the two groups. As expected, the model for end-stage liver disease and sodium (MELD-Na) score was significantly higher in the AKI group than in the non-AKI group; however, the retention rate of indocyanine green 15 min after administration (ICG-R15), which was examined only in non-transplant patients, showed no difference between the two groups. The proportion of diseases requiring operation showed a significant difference: hepatocellular carcinoma was the most prevalent disease in both groups; liver cirrhosis requiring transplantation was more common in the AKI group; and liver metastasis of other primary cancers was present in >30% of patients in the non-AKI group. Likewise, liver transplantation was performed more frequently and the duration of operation was significantly longer in the AKI group than in the non-AKI group (Table 3). However, blood loss during the operation or the intraoperative and postoperative 24-h fluid balance showed no difference between the two groups, whereas more patients with AKI needed furosemide injection than those without AKI (Table 3). We also analyzed the characteristics of patients with subclinical versus clinical AKI (Additional file 1: Table S1). Patients with subclinical AKI had higher albumin, lower AST level, shorter operation time, and less use of furosemide than patients with clinical AKI. Only 19.0% (8 of 42) patients with subclinical AKI compared to 80.0% (8 of 10) patients with clinical AKI had transplantation.

**Table 2:** **Baseline characteristics**

	AKI group (n =52)	Non-AKI group (n =79)	P
Preoperative (baseline)			
Male (%)	35 (67.3)	47 (59.5)	0.461
Age, years	59.06 ± 10.39	55.43 ± 13.47	0.085
Hypertension (%)	18 (34.6)	26 (32.9)	0.852
Diabetes (%)	15 (28.8)	17 (21.5)	0.407
Heart disease (%)^1)^ (EF < 50% or coronary artery disease)	2 (3.8)	2 (2.5)	0.649
Baseline eGFR, ml/min/1.73 m^2^	94.94 ± 25.41	95.46 ± 20.68	0.898
MELD-Na score	11.46 ± 7.06	8.05 ± 3.11	0.002
Child-Pugh score	6.21 ± 2.18	5.29 ± 1.04	0.006
ICG-R15,%^2)^	12.79 ± 9.36	12.22 ± 5.96	0.743
Mean arterial pressure, mmHg	86.38 ± 10.19	88.00 ± 9.65	0.359
Total bilirubin, mg/dL	2.27 ± 4.25	0.88 ± 1.80	0.030
Serum Na, mmol/L	138.33 ± 6.01	140.22 ± 2.16	0.034
Prothrombin time, INR	1.23 ± 0.43	1.048 ± 0.23	0.006
Albumin, g/dL	3.70 ± 0.58	3.97 ± 0.55	0.009
ALT, IU/L	25.85 ± 15.10	28.47 ± 40.10	0.653
AST, IU/L	35.15 ± 16.22	30.28 ± 25.18	0.218
CRP, mg/L	6.56 ± 16.40	10.33 ± 35.99	0.503
Reason for operation (%)			<0.001
Hepatocellular carcinoma	29 (55.8)	25 (31.6)	
Liver metastasis	3 (5.8)	24 (30.4)	
Cholangiocarcinoma	9 (17.3)	10 (12.7)	
Liver cirrhosis	7 (13.5)	2 (2.5)	
Others	4 (7.7)	18 (22.8)	

^1)^Ejection fraction <50% or coronary artery disease, ^2)^Measured only in patients with no liver transplantation.

**Table 3:** **Operation-related characteristics**

	AKI group (n =52)	Non-AKI group (n =79)	P
Duration, min	483.27 ± 274.72	366.20 ± 187.84	0.009
Fluid balance, mL	2425 ± 3024	1696 ± 2342	0.145
Blood loss, mL	431 ± 1070	303 ± 487	0.353
Postoperative 24-h fluid balance, mL	872 ± 1523	1135 ± 1521	0.335
Use of furosemide (%)	16 (30.8)	9 (11.4)	0.011
Types of operation (%)			<0.001
Hepatectomy	15 (28.8)	40 (50.6)	
Hepatectomy + other operation	21 (40.4)	35 (44.3)	
Liver transplantation	16 (30.8)	4 (5.1)	

### Risk factors for the development of AKI

Next, we performed multivariate logistic regression analysis to determine the preoperative risk factors for the development of clinical and subclinical AKI. The preoperative variables included were as follows: age, sex, hypertension, diabetes mellitus, preoperative albumin and ALT levels, and MELD-Na score. We identified that older age and higher MELD-Na score were independent predictors for the development of postoperative AKI (Table 4).

**Table 4:** **Preoperative risk factors for the development of AKI**

	Odds ratio	95% CI	P
Age	1.040	1.005 – 1.076	0.024
MELD-Na score	1.184	1.071 – 1.308	0.001

### Postoperative changes of eGFR and outcome measures

One hundred and ten (84.0%) patients completed 6-month follow-up visits and laboratory tests. Table 5 presents the changes in eGFR from baseline to 6 months postsurgery and the other outcome measures. Whereas eGFR levels at 1 and 3 months postsurgery were not different, eGFR in the AKI group at 6 months postsurgery was significantly lower than that in the non-AKI group (p =0.007). The postoperative hospital stay was also significantly longer in the AKI group. However, there was no difference in mortality between the two groups during the 6-month follow-up period.

**Table 5:** **Changes of eGFR during 6 months of follow-up and outcomes**

	AKI group	Non-AKI group	P
eGFR			
Baseline	94.94 ± 25.41	95.46 ± 20.68	0.898
At discharge	95.03 ± 36.34	105.69 ± 24.99	0.049
1 month	89.74 ± 28.38	95.79 ± 19.40	0.158
3 months	86.46 ± 26.32	89.87 ± 18.82	0.413
6 months	79.61 ± 18.22	88.97 ± 16.88	0.007
Hospital stay after surgery, days	22.4 ± 22.9	13.0 ± 10.1	0.007
Death during 6 months	3 (5.8%)	2 (2.5%)	0.385

Significant differences in the 6-month eGFR and hospital stay were still noted when only patients with subclinical AKI and those without AKI were compared (eGFR at 6 months, 80.62 ± 16.07 versus 88.97 ± 16.88 ml/min/1.73 m^2^, p =0.020; hospital stay, 17.2 ± 11.6 versus 13.0 ± 10.1 days, p =0.047). However, there was no difference in mortality between the two groups (Table 6).

**Table 6:** **Comparison of outcomes in the non-AKI, subclinical AKI, and clinical AKI groups**

	Non-AKI (n =79)	Subclinical AKI (n =42)	Clinical AKI (n =10)
Baseline eGFR	95.46 ± 20.68	90.96 ± 21.86	98.34 ± 12.35
eGFR at 6 months	88.97 ± 16.88	80.62 ± 16.07*	76.24 ± 24.81*
Hospital stay after surgery	13.0 ± 10.1	17.2 ± 11.6*	44.1 ± 41.4*
Death during 6 months	2 (2.5%)	3 (7.1%)	0 (0%)

*P <0.05 compared with the non-AKI group. Outcomes were not significantly different between patients with subclinical AKI and those with clinical AKI.

### Predictors of 6 months renal outcome

We evaluated the parameters independently associated with the degree of changes in eGFR at 6 months postsurgery (ΔeGFR = eGFR at 6 months – baseline eGFR) by multiple linear regression analysis. The maximum level of urine NGAL (p =0.011), a positive intraoperative fluid balance (p =0.027), and having liver transplantation (p =0.012) were significantly and negatively associated with ΔeGFR after adjustment for age, sex, hypertension, diabetes, MELD-Na score, and length of operation (Table 7).

**Table 7:** **Multiple linear regression analysis of the degree of changes in eGFR 6 months post surgery**

	B	Standard error	Beta	P	95% CI
Constant	-6.288	9.963		0.529	-26.054 to 13.478
Age	0.042	0.145	0.026	0.771	-0.245 to 0.329
Sex	2.591	3.196	0.065	0.420	-3.750 to 8.931
Hypertension	4.104	3.635	0.098	0.262	-3.108 to 11.315
Diabetes	-0.649	3.871	-0.014	0.867	-8.329 to 7.031
MELD-Na score	0.150	0.390	0.043	0.702	-0.624 to 0.924
Fluid balance during operation	-0.002	0.001	-0.219	0.027	-0.003 to 0.000
Length of operation	-0.003	0.010	-0.035	0.768	-0.022 to 0.016
Urine NGAL, max.	-0.016	0.006	-0.221	0.011	-0.028 to -0.004
Liver transplantation	-18.830	7.315	-0.358	0.012	-33.343 to -4.318

F =6.888, R^2^ = 0.383, P <0.001.

## Discussion

AKI is an important postoperative complication that increases morbidity and mortality, with an incidence ranging from 10% to 30% after a major surgery [5, 6]. In a retrospective study of 10,518 patients with normal renal function, postoperative AKI was associated with increased mortality even in patients who recovered their renal function at the time of hospital discharge [5]. In another study, patients with postoperative AKI were reported to have more dependency in the activities of daily living at 6 months after discharge [6].

In contrast to cardiac or vascular surgery, the incidence or risk factors of AKI after intraperitoneal surgery are not well known due to the paucity of data. In our study population of patients with a baseline eGFR of ≥60 ml/min/1.73 m^2^ who underwent elective hepatobiliary surgery, 7.6% developed AKIN criteria-based clinical AKI but none needed RRT. The overall incidence and severity of AKI were much lower than those in a previous report that demonstrated an incidence of AKI of 15.1% after hepatic resection [7]. Although direct comparison of the patients’ baseline and operative characteristics is difficult, the higher prevalence of chronic kidney disease (CKD) (12.8%) in the previous study might be one reason for this discrepancy.

We next tested the validity of serum and urine NGAL in the early detection of AKI. NGAL is the most widely studied biomarker for AKI; however, most studies have been performed in patients undergoing cardiac surgery or transplantation [8–13]. As expected, we found that urine NGAL obtained at 12 h postsurgery was the most predictive parameter for the detection of AKI, with a cutoff value of 92.85 ng/mL. Serum NGAL did not show predictive value in our population partly because it can rise in liver injury. Organs other than kidney, such as liver, lung, and gastrointestinal tract, also express NGAL under physiologic condition and the expression is increased under injured or post-operative condition [14]. Our previous study with critically ill patients and another study with liver transplant patients also presented the usefulness of urinary NGAL compared to plasma NGAL in the prediction of AKI or prognosis [15, 16]. Although another study performed in patients undergoing cardiac surgery presented a similar value (87 ng/mL) [11], the optimal cutoff values for detecting postoperative AKI vary across studies. In addition, defining the proper time point for sample collection is also important. Several previous studies performed in patients undergoing cardiac surgery or liver transplantation suggest that NGAL levels at 0–6 h postsurgery are meaningful for the early detection of AKI [8–11, 13, 17], whereas a study performed in patients undergoing non-cardiac major surgery showed no correlation of NGAL levels at 2 or 6 h postsurgery with the development of AKI [18]. In our study, we also observed that urine NGAL-12 h, but not NGAL-6 h, reliably predicts clinical AKI in patients undergoing hepatobiliary surgeries. Concerning the proper cutoff value or time point, further larger studies with different types of surgeries are needed.

Recent advances in biomarker research have made it possible to identify patients who showed only increased levels of tubular injury markers without fulfilling the consensus criteria for AKI. Those patients could not have been recognized in the pre-biomarker era because their urine output and serum creatinine levels are well maintained. However, several recent studies demonstrated that patients with this type of subclinical AKI comprises 15-20% of patients admitted to an intensive care unit or an emergency department, and they have increased risk of death or need for dialysis compared with patients without elevated levels of tubular damage markers [2, 19]. In our study, with an NGAL cutoff value of 92.85 ng/mL, >30% of patients who underwent elective hepatobiliary surgery developed subclinical AKI. We then included these patients in the AKI group (n =52, 39.7%) and compared their preoperative risk factors with those of the non-AKI group.

Among several factors that were significantly different between the AKI and non-AKI groups in univariate analysis, older age and higher MELD-Na score were found to be independent preoperative risk factors for AKI after hepatobiliary surgery. Although several models for predicting AKI after cardiac surgery, such as Continuous Improvement in Cardiac Surgery Study and Cleveland Clinic Score, have been proposed and validated extensively [20], only few studies developed a prediction model for AKI in non-cardiac surgeries [7, 21]. Kheterpal et al., by reviewing 152,244 operations, identified 11 independent preoperative predictors of AKI after general surgeries: age ≥56 years, male sex, emergency surgery, intraperitoneal surgery, diabetes mellitus necessitating oral or insulin therapy, active congestive heart failure, ascites, hypertension, and mild to moderate preoperative renal insufficiency [21]. More recently, Slankamenac et al. developed and validated an AKI prediction score system in patients undergoing liver resection, similar with our study. In that study, they noted that postoperative AKI developed in 15.1% and that preoperatively elevated ALT level, preexisting cardiovascular disease, chronic renal failure, and diabetes were the strongest predictors of AKI defined by the RIFLE criteria [7]. Although our study did not aim to develop a prediction model for postoperative AKI and had a small sample size for validating a new model, we could identify that old age and high MELD-Na score were significant risk factors for postoperative AKI after hepatobiliary surgery. On the other hand, ALT or the presence of diabetes was not associated with the development of postoperative AKI in our study. The major differences between our study and Slankamenac et al.’s were that we excluded patients with CKD or those who underwent emergency surgeries and included patients with subclinical AKI. These results could suggest that in patients without CKD, age and the degree of underlying liver dysfunction might be more important in the development of postoperative AKI.

It is noteworthy that preoperative MELD-Na score that includes the INR of prothrombin time, and bilirubin, serum creatinine, and Na levels can be considered risk factors for postoperative AKI even in patients with normal renal function. No study to date has investigated the predictive role of the scoring system in postoperative complications. The MELD incorporating serum sodium, known as “MELD-Na”, was developed for the outcome prediction of cirrhosis and is considered to have better prognostic ability than the Child-Pugh classification or the MELD [22, 23]. In addition, the MELD-Na score has also been reported to be significantly related to survival in patients with hepatocellular carcinoma or pancreatic and gastric cancer-related ascites [24–26]. Similarly, we also observed that a high preoperative MELD-Na score was independently associated with the development of AKI after hepatobiliary surgery. We chose the MELD-Na score instead of the MELD score because we observed that the Na level was significantly lower in the AKI group in univariate analysis, in addition to the significantly prolonged prothrombin time (INR), elevated serum bilirubin level, and lower albumin level.

Interestingly, we assigned patients with subclinical AKI to the AKI group and compared the outcomes. While dialysis requiring AKI is a well-known factor of poor outcome of AKI such as longer hospital stay, higher mortality, and predisposition to end-stage renal disease, we observed in our study that patients with stage 1, 2, or 3 without the need for RRT or even those with subclinical AKI had significantly longer hospital stay and worse 6 months eGFR than the non-AKI group. The difference was also confirmed by a separate analysis between patients with subclinical AKI and the non-AKI group patients. To the best of our knowledge, this is the first study that demonstrated that even subclinical postoperative AKI might portend poor long-term renal outcome. Although our patients with subclinical AKI still had an eGFR of 80 ml/min/1.73 m^2^ after 6 months of observation, further studies with a longer follow-up period would show a larger impact on patients’ renal outcome. Moreover, the maximum level of urine NGAL, which was used for the diagnosis of subclinical AKI, was demonstrated to be independently associated with the extent of loss of renal function at 6 months postsurgery, even after the adjustment for other variables including liver transplantation.

All these findings in our study could strengthen the important role of urine NGAL as a sensitive biomarker and an outcome predictor of postoperative AKI, and also emphasize the need for recognizing and evaluating patients who develop subclinical AKI.

Along with urine NGAL, having liver transplantation was also demonstrated to be independently associated with poor long-term postoperative renal outcome. In addition to the possibility that frequent development of AKI after liver transplantation leads to worse renal outcome, another possibility for worse 6 month renal outcome in liver transplantation might include the unavoidable use of immunosuppressive drugs such as calcineurin inhibitors. Another factor that was independently associated with worse 6 months renal outcome was a positive fluid balance during the operation. Previous reports also highlighted the importance of fluid overload as a risk factor for death and lack of renal recovery in patients with AKI; therefore, restricted perioperative fluid administration was recommended to reduce postoperative complications and mortality [27–33]. Our study, by demonstrating worse long-term renal outcome, reaffirms the negative impact of positive fluid balance in hepatobiliary surgeries. This association was still significant even after the adjustment for liver transplantation, age, or MELD-Na score.

Despite several novel findings, this study has some limitations. It is a single-center prospective study, and its small sample size restricted the development or validation of a model for predicting postoperative AKI or long-term renal outcome. In addition, we only enrolled patients with normal renal function and only four patients had heart diseases, which may limit the general application of the results of this study. We excluded patients with eGFR <60 ml/min/1.73 m^2^ because decreased eGFR can be a confounding factor in interpreting the incidence of post-operative AKI and the 6 months renal outcome post surgery. Inclusion of patients irrespective of the type of hepatobiliary surgery, including liver transplantation, might also be a limitation. However, we adjusted for liver transplantation when analyzing the risk factors for 6 months renal outcome. Furthermore, as already mentioned, more frequent follow-up examinations of serum or urine NGAL levels may show additional information, although urine NGAL-12 h was the most predictive parameter for determining AKI in our patients. Finally, we observed the patients for only 6 months. Although our study identified several important findings, these results need to be further validated in a larger cohort of patients with a longer follow-up period.

## Conclusions

In conclusion, 7.6% and 32.1% of patients with normal baseline renal function undergoing elective hepatobiliary surgery developed clinical and subclinical AKI, respectively. Older age and higher MELD-Na score were risk factors for postoperative AKI. Urine NGAL was demonstrated to be useful both for the early diagnosis of postoperative AKI and for the prediction of 6 months renal outcome. In addition, liver transplantation and positive intraoperative fluid balance as well as urine NGAL were associated with poor 6 months renal outcome. Further studies of postoperative AKI in patients undergoing different types of surgeries or with different morbidities are needed to validate the role of biomarkers and find predictors of outcomes.

## Electronic supplementary material

Additional file 1: Table S1: Baseline and operation-related characteristics of subclinical versus clinical AKI group. (DOC 52 KB)
