# Supplementary material for: The incidence and risk factors of acute kidney injury after hepatobiliary surgery: a prospective observational study
Source: BMC Nephrol. 2014 Oct 23;15:169. doi: 10.1186/1471-2369-15-169 (PMC4221681; doi:10.1186/1471-2369-15-169)
Supplement: Supplementary file 1 — Additional file 1: Table S1: Baseline and operation-related characteristics of subclinical versus clinical AKI group. (DOC 52 KB) [file 12882_2014_859_MOESM1_ESM.doc]

**Additional file 1: Table S1**. Baseline and operation-related characteristics of subclinical versus clinical AKI group

|  | Subclinical AKI  (n=42) | Clinical AKI  (n=10) | P |
| --- | --- | --- | --- |
| Preoperative (baseline) | | | |
| Male (%) | 28 (66.7) | 7 (70.0) | 1.000 |
| Age, year | 59.60 ± 10.63 | 56.80 ± 9.51 | 0.450 |
| Hypertension (%) | 14 (33.3) | 4 (40.0) | 0.723 |
| Diabetes (%) | 12 (28.6) | 3 (30.0) | 1.000 |
| Heart disease (%)  (EF<50% or coronary artery disease) | 2 (4.8) | 0 (0) | 1.000 |
| Baseline eGFR, ml/min/1.73m^2^ | 90.96 ± 21.86 | 98.34 ± 12.35 | 0.311 |
| MELD-Na score | 10.19 ± 5.45 | 16.80 ± 10.40 | 0.080 |
| Child-Pugh score | 5.86 ± 1.69 | 7.70 ± 3.30 | 0.117 |
| ICG R15,% * | 12.99 ± 9.48 | 9.25 ± 8.84 | 0.590 |
| Mean arterial pressure, mmHg | 86.51 ± 10.37 | 85.83 ± 9.90 | 0.853 |
| Total billirubin, mg/dL | 1.40 ± 2.48 | 5.91 ± 7.49 | 0.091 |
| Serum Na, mmol/L | 139.33 ± 3.50 | 134.10 ± 11.15 | 0.175 |
| Prothrombin time, INR | 1.14 ± 0.28 | 1.61 ± 0.70 | 0.065 |
| Albumin, g/dL | 3.79 ± 0.54 | 3.35 ± 0.63 | 0.032 |
| ALT, IU/L | 23.81 ± 10.27 | 34.40 ± 26.64 | 0.246 |
| AST, IU/L | 31.62 ± 12.14 | 50.00 ± 22.72 | 0.032 |
| CRP, mg/L | 7.04 ± 18.17 | 4.55 ± 3.77 | 0.687 |
| Cause of operation (%)  Hepatocellular carcinoma  Liver metastasis  Cholangiocarcinoma  Liver cirrhosis  others | 25 (59.5)  3 (7.1)  7 (16.7)  3 (7.1)  4 (9.5) | 4 (40.0)  0 (0)  2 (20.0)  4 (40.0)  0 (0) | 0.068 |
| Operation related | | | |
| Duration, min | 421.07 ± 233.29 | 744.50 ± 293.03 | <0.001 |
| Fluid balance, mL | 2105 ± 2483 | 3768 ± 4612 | 0.295 |
| Blood loss, mL | 337 ± 568 | 830 ± 2191 | 0.497 |
| Postoperative 24hr fluid balance, mL | 1035 ± 1337 | 185 ± 2085 | 0.113 |
| Use of furosemide (%) | 9 (21.4) | 7 (70.0) | 0.006 |
| Types of operation (%)  Hepatectomy  Hepatectomy + other operation  Liver transplantation | 15 (35.7)  19 (45.2)  8 (19.0) | 0 (0)  2 (20.0)  8 (80.0) | 0.001 |

*This is measured in patients only with non-liver transplantation.
